# Skewed electronic band structure induced by electric polarization in ferroelectric BaTiO_3_

**DOI:** 10.1038/s41598-020-67651-w

**Published:** 2020-07-01

**Authors:** Norihiro Oshime, Jun Kano, Eiji Ikenaga, Shintaro Yasui, Yosuke Hamasaki, Sou Yasuhara, Satoshi Hinokuma, Naoshi Ikeda, Pierre-Eymeric Janolin, Jean-Michel Kiat, Mitsuru Itoh, Takayoshi Yokoya, Tatsuo Fujii, Akira Yasui, Hitoshi Osawa

**Affiliations:** 1 Graduate School of Natural Science and Technology, Okayama University, Okayama, 700-8530 Japan; 2 Japan Science and Technology Agency, PRESTO, Kawaguchi, Saitama 332-0012 Japan; 3 Université Paris-Saclay, CentraleSupélec, CNRS, Laboratoire SPMS, 91190 Gif-sur-Yvette, France; 4 Japan Synchrotron Radiation Research Institute, JASRI, Sayo, Hyogo 679-5198 Japan; 5 Laboratory for Materials and Structures, Tokyo Institute of Technology, Yokohama, 226-8503 Japan; 6 Laboratory for Advanced Nuclear Energy, Tokyo Institute of Technology, Tokyo, 152-8550 Japan; 7 Innovative Oxidation Team, Interdisciplinary Research Center for Catalytic Chemistry, National Institute of Advanced Industrial Science and Technology, Tsukuba, Ibaraki 305-8565 Japan; 8 Research Institute for Interdisciplinary Science, Okayama University, Okayama, 700-8530 Japan

**Keywords:** Ferroelectrics and multiferroics, Ferroelectrics and multiferroics

## Abstract

Skewed band structures have been empirically described in ferroelectric materials to explain the functioning of recently developed ferroelectric tunneling junction (FTJs). Nonvolatile ferroelectric random access memory (FeRAM) and the artificial neural network device based on the FTJ system are rapidly developing. However, because the actual ferroelectric band structure has not been elucidated, precise designing of devices has to be advanced through appropriate heuristics. Here, we perform angle-resolved hard X-ray photoemission spectroscopy of ferroelectric BaTiO_3_ thin films for the direct observation of ferroelectric band skewing structure as the depth profiles of atomic orbitals. The depth-resolved electronic band structure consists of three depth regions: a potential slope along the electric polarization in the core, the surface and interface exhibiting slight changes. We also demonstrate that the direction of the energy shift is controlled by the polarization reversal. In the ferroelectric skewed band structure, we found that the difference in energy shifts of the atomic orbitals is correlated with the atomic configuration of the soft phonon mode reflecting the Born effective charges. These findings lead to a better understanding of the origin of electric polarization.

## Introduction

A spontaneous electric polarization of ferroelectric perovskite-type oxides originates in the relative ionic displacement of metal and oxygen ions, with inversion symmetry breaking. The electric field generated by the electric polarization causes an electrostatic potential gradient along the polarization direction in ferroelectric materials^1,2^, forming a skewed band structure. Such a gradual potential influence on the energy levels of atomic orbitals drives rectification of electron transfer in FTJs^3–9^. FTJs consist of two different metals separated by a ferroelectric thin film, i.e. metal (M)–ferroelectric (FE)–M junctions. The barrier with an electrostatic potential gradient formed by ferroelectric allows electron tunneling^3,4^. The polarization reversal can switch the gradient orientation producing a large tunneling electroresistance (TER) effect at M–FE–M junctions^5^. Recently, a TER effect with a magnitude of 10^4^ has been demonstrated in M–FE–heavily doped semiconductor (hS) junctions, where the hS was Nb-doped SrTiO_3_ (NSTO)^6–8^. At the FE-hS interface, the NSTO creates variable depletion and accumulation states controlled by polarization reorientation^8^. In FTJs, the orientation of electric polarization controlled by the external electric field can present 1 or 0 in Boolean algebra used in nonvolatile random access memory, i.e. FeRAM with high read and write speeds^10^. FTJs also show a memristive function as a tunable resistive behavior utilizing the artificial neural network^11,12^. Since band engineering of the FE–hS interface can improve the effective electron transfer, FTJs are currently seen as a promising heterostructure, one of the several advanced ferroelectric functional devices.

Electronic structures modulated by electric polarization yield a so-called ferroelectric band skewing (FEBS) structure. Although gradual band structures can be described by the effect of electric polarization on ferroelectric materials, the common gradual band phenomenon has been discussed as an interfacial effect in a pn junction, which consists of non-polar semiconductors such as Si and GaAs^13^. Since the band “bending” structure in a pn junction is derived from the different work functions of two semiconductors, it cannot be reversed by any external field. In contrast, the ferroelectric band “skewing” structure is made possible by reversible electrical polarization. Ferroelectricity is thus a key function for future electronic devices using a FEBS structure. The bent-band structure of pn junctions has been observed by transmission electron microscopy^14^; however, the actual occurrence of FEBS remains unproven experimentally. One of the crucial problems that need to be further clarified is the fact that the actual location of the FEBS is not known^15–17^. Therefore achieving the fundamental understanding for the energy shift of atomic orbitals in terms of their depth profile is an important basis for understanding the electronic structure in ferroelectrics. In the present study, angle-resolved hard X-ray photoemission spectroscopy (AR-HAXPES) is used to allow for the direct observation of the FEBS structure in ferroelectric materials. AR-HAXPES with synchrotron radiation has the advantage to provide a 20-nm-deep profile of photoelectron emission spectra^18^. Our results show that electronic core levels and valence band shift to a higher energy in accordance with the orientation of the electrical polarization in ferroelectric BaTiO_3_ (BTO) thin films. The FEBS structure shows a linear potential slope, which is a bulk-like feature. But this slope is suppressed at the surface and interface. We also demonstrate that this FEBS structure can be flipped through polarization reversal. In the valence band and core-level atomic orbitals, the binding energy of covalent states shifts more than that of non-covalent states. This behavior is attributed to the correlation with phonon oscillations, which leads to a straightforward understanding of the origin of electrical polarization in the viewpoint of the Born effective charges.

## Results

**Figure 1:**
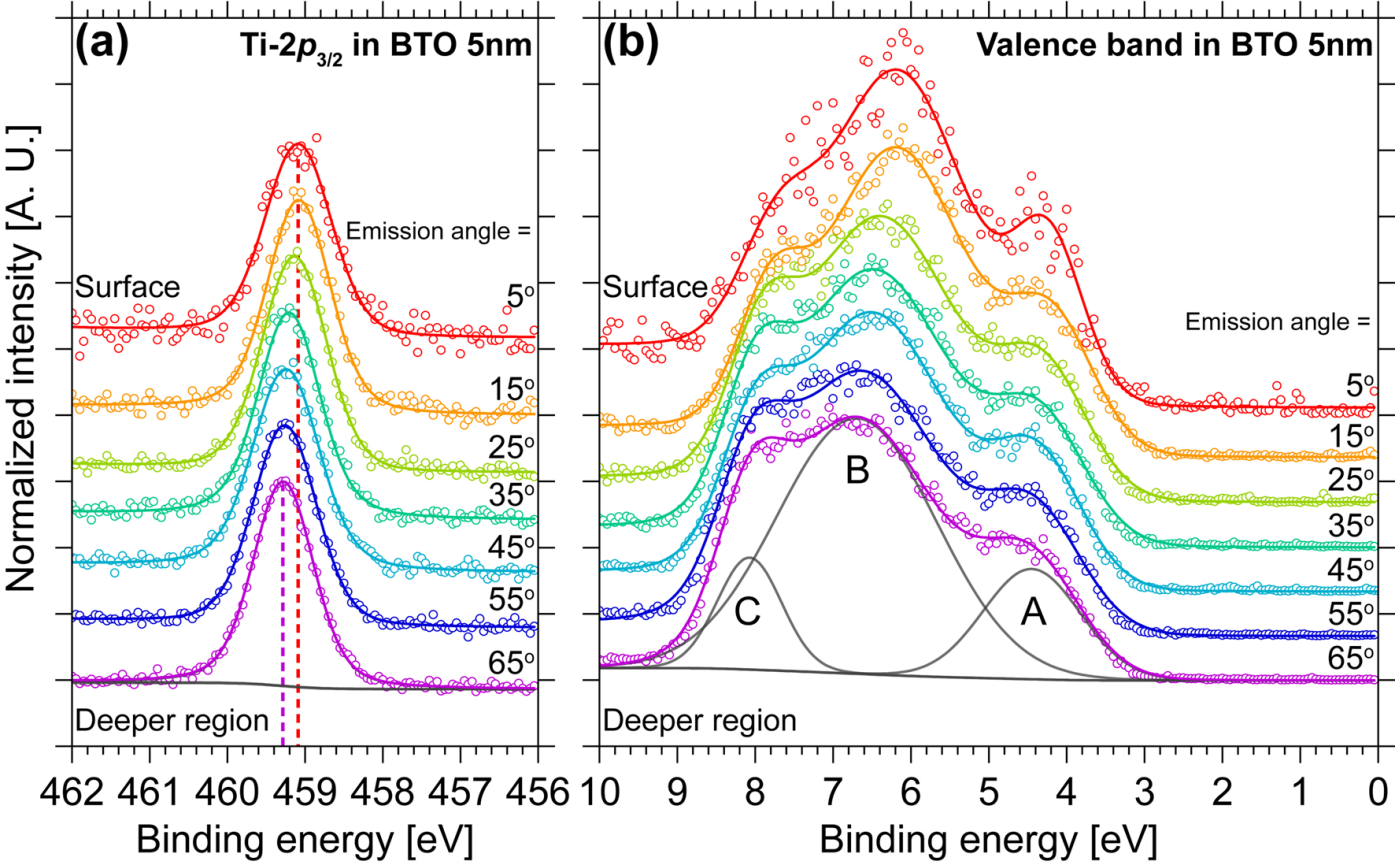
AR-HAXPES spectra observed at each emission angle: (**a**) Ti-2*p*_3/2_ and (**b**) valence band in the 5 nm thick BTO. Red and purple circles are surface and deeper regions, respectively. In the spectrum at emission angle = 65°, curves of background and Voigt function are drawn as gray lines.

The ferroelectric BTO thin films of AR-HAXPES was carried out at BL47XU in SPring-8. The detailed experimental setup is described in the Supplementary Information. Figure 1a shows AR-HAXPES spectra of Ti-2*p*_3/2_ in 5 nm thick BTO observed at various depths. The peak shifts to a higher binding energy region with increasing escape depth. Valence band shows similar behavior to core-level binding-energy shift (Fig. 1b). The valence band consists of three electronic states: one pure O-2*p* orbital and two O-2*p* and Ti-3*d* hybridized states are called as regions A, B and C, respectively^19,20^. This is the first observation of the depth profiles of the electronic structure of ferroelectrics.

**Figure 2:**
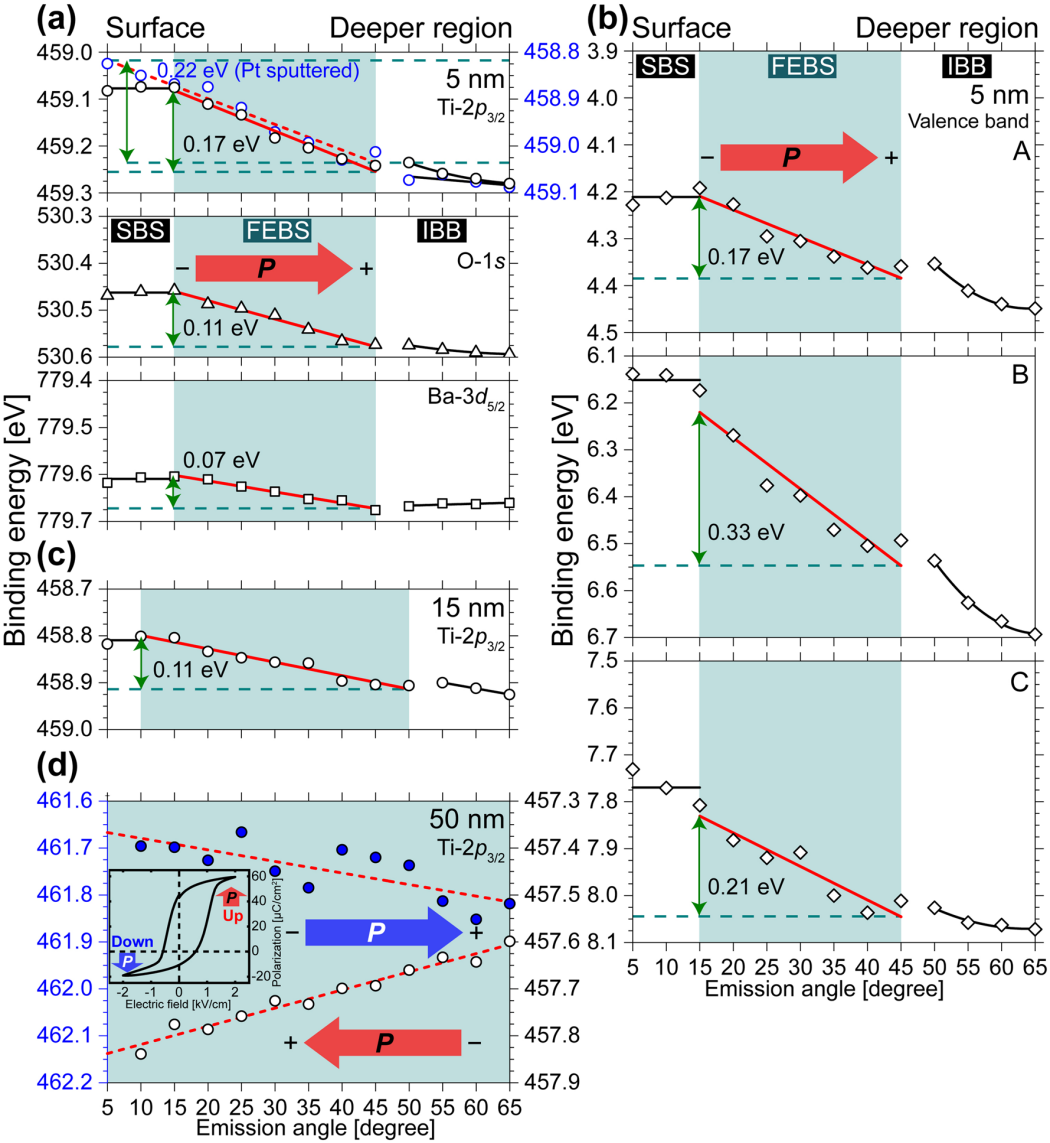
Depth dependence of binding energies of BTO: (**a**) Ti-2*p*_3/2_, O-1* s*, Ba-3*d*_5/2_ in 5 nm thickness, (**b**) valence band in 5 nm, (**c**) Ti-2*p*_3/2_ in 15 nm, and (**d**) Ti-2*p*_3/2_ in 50 nm. Plots indicate the peak energy estimated by the center position of FWHM at each emission angle. Solid and dashed red lines are obtained by a linear function fit. Green arrows show the energy shift in FEBS. Red arrows indicate the inherent direction of electric polarization. Blue arrow seen in (**d**) indicates the direction of electric polarization by switching induced by the applied electric field. The inset graph in (d) is the *P*–*E* loop of the 50 nm sample.

For a quantitative discussion of the energy shift in atomic orbitals, we fitted the spectra by employing superposition of Voigt functions (see Section S4, Supplementary Information). Figure 2 shows the depth-dependence of binding energies of core levels and valence band in BTO. In the 5 nm-thick BTO sample without top electrode as seen in Fig. 2a, b, all atomic orbitals have two inflection points at emission angles of 15° and 45°. We observe that for the emission angles between 15° and 45°, the binding energy increases monotonically with increasing depth in the BTO. This energy shift behavior is consistent with a potential slope where the electric polarization points into the NSTO substrate as confirmed by the piezoresponse phase images (see Supplementary Fig. S1d, e online). Thus we can assign that this slope appears in the internal layer as FEBS induced by electric polarization. We have investigated the thickness dependence of FEBS slope. The energy shifts of Ti-2*p*_3/2_ core level in BTO with a thickness of 5 and 15 nm are 0.17 and 0.11 eV, respectively (see Fig. 2a, c). According to ref.^21^, a remanent polarization *P*_r_ has values of 12 and 26 µC/cm^2^ with 5 and 15 nm-thick BTO, respectively. Since an electrostatic potential *V* (eV) is proportional to *q*/*r*, we compared *V*/(*q*/*r*) in the 15 nm thick BTO to that in 5 nm BTO. Here *q* (C) and *r* (m) stand for charges formed by the electric polarization and the film thickness, respectively. The parameter *q* is estimated by the product of *P*_r_ and the area of the impact point of the incoming x-ray beam (30 × 40 μm^2^). Obtained *V*/(*q*/*r*) are good agreement with each other (5.9 for 5 nm and 5.3 for 15 nm). Thus, we concluded that the slope in the internal layer is FEBS. Strong depolarizing field often induces polydomain structures in thin films^22,23^. Despite the absence of top electrodes, the monodomain structure of our samples was confirmed as seen in Supplementary Fig. S1 online. We concluded that the potential slope induced by electric polarization has been observed as FEBS with AR-HAXPES. At the surface (corresponding to emission angles 5°–15°), the energy shift is suppressed. This behavior implies the occurrence of polarization reduction caused by surface relaxation even though the polarization in thin films is maintained by the epitaxial strain^24^. The flat surface-potential then appears and shall be referred to as the surface band skewing (SBS). When a top electrode is deposited on the BTO, the surface polarization can be sufficiently screened and stabilized, and as result the energy shift increases. We observed the disappearance of SBS and increase of FEBS slope in Pt sputtered BTO (see red dashed line in Fig. 2a). The interface between BTO thin film and NSTO substrate is determined by the second inflection point between emission angles of 45° and 50°. In the deeper region of BTO corresponding to emission angles 45°–65°, the slope of energy shift shows non-linearity compared to the internal layer. This can be attributed to the lattice mismatch that occurs at the interface causing a strong strain. It induces a small energy shift at the interface between the BTO film and its substrate. This modulated FEBS is interpreted as an interfacial band bending (IBB). Valence band shows a similar behavior to the core levels (Fig. 2b).

Next, we demonstrate that the FEBS can change its slope by the switching of polarization as shown in Fig. 2d. Our result experimentally proves the ferroelectric origin of the band-skewing structure, which corresponds to the theoretical prediction^9^. Note that the energy shift of BTO with 50 nm thickness is larger than the one measured for thinner samples of 5 and 15 nm. This is attributed to the different bottom electrode and substrate. 50 nm sample seen in Fig. 2d used Pt and SrRuO_3_ (SRO) for the electrodes, which is different from the other samples such as 5 and 15 nm (Our samples condition is described in the “Methods”). Pt and SRO have a work function of 5.65 and 5.21 eV, respectively^25–27^. In this case, two electrodes form the built-in field in the BTO thin film^10^, causing the additional band skewing. Moreover, we note the difference of the substrate. (LaAlO_3_)_0.3_–(SrAl_0.5_Ta_0.5_O_3_)_0.7_ (LSAT) single crystal used as a substrate for 50-nm sample has a smaller lattice constant (*a* = 3.87 Å) than Nb 0.5 wt% doped NSTO (*a* = 3.91 Å) inducing an in-plane compressive strain^28,29^. This strain increases the tetragonality of BTO and enhances the electric polarization. We measured a *P*–*E* loop resulting in a large *P*_r_ ~ 43 μC/cm^2^ (see the inset of Fig. 2d). This value is larger than the bulk one. In the bulk or similar state with large *P*_r_, a depolarizing field will be proportional to *P*_r_. Since the slope of FEBS reflects both the built-in field and depolarizing field, the large slope observed in the 50-nm thick sample can be attributed to the work function and compressive strain. For 5- and 15-nm samples, the built-in field partially affects the slope of FEBS.

Since a screening efficiency at the M–FE interface plays an essential role in the understanding of polarization stability and the developing FTJ devices, we estimated an effective screening length λ_eff_ and a depolarizing field ${\mathcal{E} }_{d}$ using our experimental values. Generally, the ${\mathcal{E} }_{d}$ in ferroelectric thin films increases as thickness decreases. A small λ_eff_ means a better screening of these surface charges. First, we estimated λ_eff_ from the formula $\Delta V=\left({\lambda }_{eff}/{\epsilon }_{0}\right)\cdot P$, where $\Delta V$, ${\epsilon }_{0}$, and $P$ stand for the voltage drop in a short-circuited M-FE-M, the permittivity of free space, and spontaneous polarization, respectively^28^. In the present study, we used the energy shift of FEBS as $\Delta V$ and *P*_r_ as *P*. The values of *P*_r_ in 5 and 15 nm BTO are taken from Ref.^21^. In the case of a 50-nm thick sample, we used our *P*–*E* loop data as seen in the inset of Fig. 2d. And then, we calculated ${\mathcal{E} }_{d}$ as ${\mathcal{E} }_{d}=-2\Delta V/d$, where *d* is the film thickness. As shown in Table 1, the values of λ_eff_ and ${\mathcal{E} }_{d}$ are a good agreement with the first-principles calculation results^30,31^. The film thickness dependence appears in both λ_eff_ and ${\mathcal{E} }_{d}$ of the 5- and 15-nm samples. Although the value of λ_eff_ of the 5-nm sample (0.13 Å) is smaller than the first-principle result of the BTO/SRO interface (0.23 Å)^30^, this difference may be attributed to the different electrodes, albeit it remains in the same order of magnitude. In the 50-nm sample, we found a small ${\mathcal{E} }_{d}$ value compared to thinner films. This result indicates that the 5- and 15-nm samples have the typical nature of ferroelectric thin films, which displays a significantly reduced polarization but remains ferroelectric. The 50-nm sample exhibits *q* different behavior in each direction of the electric polarization owing to the imprint^10^.

**Table 1:** Measured binding energy shift of FEBS by AR-HAXPES and remanent polarization *P*_r_ by *P*–*E* loop and calculated effective screening length λ_eff_ and depolarizing field ${\mathcal{E} }_{d}$.

	FEBS (eV)	*P*_r_ (μC/cm^2^)	λ_eff_ (Å)	${\mathcal{E} }_{d}$ (V/cm)
5 nm BTO/NSTO	0.17	12*	0.13	− 6.80 × 10^5^
15 nm BTO/NSTO	0.11	26*	0.04	− 1.47 × 10^5^
Pt/50 nm BTO/SRO/LSAT	0.24	43	0.05	− 0.96 × 10^5^
Pt/50 nm BTO/SRO/LSAT	0.15	− 11	0.12	0.60 × 10^5^

Since *P*–*E* loop of 5 and 15 nm samples could not be observed owing to the leakage current, the values of *P*_r_ in 5 and 15 nm thickness were used in the experimental values of Ref.^21^. For 50 nm sample, two types of FEBS are listed. The values of FEBS are different between the up and down direction due to the imprint.

*Ref.^21^.

When AR-HAXPES observes the whole region from the surface to the interface between BTO and NSTO as in Fig. 2a, b, the energy level of all atomic orbitals has the three components of SBS, FEBS and IBB. Based on our band-skewing lineup as described above, we discuss a band skewing structure induced by the electric polarization i.e. FEBS. The polarization produces a gradual change of electrostatic potential in a ferroelectric crystal. Since such a potential has a slope, the binding energy increases with increasing depth in the sample, as seen in FEBS component. In FTJs, when BTO film is thin enough (~ 5 nm), electronic carriers can move in BTO along the potential slope depending on the polarization direction. In our experiment, an electronic structure skewed by the electric polarization is observed through the binding energy shift of atomic orbitals. All atomic orbitals show similar behavior, but the magnitude of the energy shift derived from FEBS is different. Ti-2*p*_3/2_, O-1* s* and Ba-3*d*_5/2_ have binding energy shift of 0.17, 0.11 and 0.07 eV, respectively (see Fig. 2a). Ti ion shows larger energy shift compared to O and Ba ions. But O ion shows slightly larger shift than Ba one. We argue that the energy shift is correlated with the Born effective charges. In accordance with Ref.^32^, the ratios of Born charge Z^*^ and nominal ionic one in each atoms are evaluated as Z^*^_Ba_/(+ 2) = 1.39, Z^*^_O_/(− 2) = 1.67, and Z^*^_Ti_/(+ 4) = 1.81 corresponding to the magnitude relationship of the energy shift. We consider this fact from the viewpoint of the lattice dynamics^33–35^. The polar phonon mode in BTO is assigned to the oscillation of a Ti ion and an oxygen octahedron; the so-called Slater mode^35^. Softening of the Slater mode induces a structural phase transition together with the generation of electric polarization. This means that the displacement of a Ba ion makes no contribution to the Slater mode. There are large binding energy shifts in Ti and O ions, which have a Slater mode atomic configuration, but Ba has a small shift. In addition, Ti and O ions are hybridized, while Ba displays ionic behavior^36^. Thus, the large binding energy shift of Ti and O ions triggers hybridization. In the valence band, regions B and C which contain covalent Ti-3*d* and O-2*p* hybridized states show larger energy shift than non-covalent region A. This valence band behavior confirms the interpretation above. Our finding that the electronic states of the soft mode atomic configuration induce band skewing is in line with previous ab initio calculations of the Born effective charges^32^. From our experimental findings, we conclude that atomic orbitals derived from the atomic configuration of soft phonon show a large binding energy shift. Although the amplitude of electric polarization has usually been evaluated by the combination of ionic displacement and covalency in ferroelectrics, the origin of electric polarization can be simply understood from the electronic band skewing structure, constituting an important advance in the ferroic science.

In summary, we have experimentally studied the electronic band skewing structure in ferroelectric BTO thin films by AR-HAXPES. Our results show that the binding energy of core levels and valence band display a shift that is depth dependent, providing the straightforward interpretation that the ferroelectric electronic structure has three band-skewing components, i.e. SBS, FEBS, and IBB. In particular, FEBS has a slope along the electric polarization direction. This band skewing structure can change its slope by switching polarization. FEBS is affected by the electronic configuration and the characteristic behavior of ions associated to a soft phonon mode. The theory of electronic structure can guide research on ferroelectrics. Our results will allow the development of novel FTJ devices using ferroelectrics.

## Methods

### Sample preparation

For AR-HAXPES measurement, epitaxial BTO thin films grown on conductive substrates were used. Because conventional ferroelectric oxides have small carrier concentrations, resulting in charge at the surface effective photoemission detection is usually difficult. The conductive substrate also works as a bottom electrode for the polarization switching. In the present study, we prepared two types of samples. One is for observation of the thickness dependence. Ferroelectric BTO thin films with 5 and 15 nm thickness were deposited on (100) Nb 0.5 wt% doped NSTO single-crystal substrates with cutting into the size of 5 × 5 × 0.5 mm^3^ by pulsed laser deposition (PLD), using the 266 nm 4th-harmonic wave of a Nd:YAG laser. 5 nm-thick Pt with 1 × 2 mm^2^ was capped on the 5 nm-thick BTO film by dc sputtering. The second set of samples is for observation of the polarization switching dependence. Pt/BTO/SRO was deposited on (100) LSAT single crystal by the same way, where the thickness of Pt, BTO and SRO are 3, 50 and 50 nm, respectively. Pt was deposited on the BTO surface as dots with the diameter of 200 μm by electron-beam evaporation. Non-ferroelectric γ-Al_2_O_3_ (ALO) thin film with 5 nm thickness on NSTO was also prepared to confirm depth profile in the heterostructure. Single-crystal substrates were washed in tetramethylammonium hydroxide aqueous solution (Semico Clean 56, Furuuchi Chemical Co.) and then rinsed in ultrapure water before thin film deposition. PLD conditions of BTO and ALO were respectively 650 and 700 °C growth temperature, 20 mTorr and 1 mTorr oxygen pressure, and 1.3 and 2.9 J/cm^2^ of laser energy. Our experiment aimed to investigate the contribution of polarization to band skewing. In this case, simple interface and single domain BTO thin film, avoiding any ferroelectric and ferroelastic domain contributions are necessary to the understanding of the observations. Crystal structures of the deposited films were confirmed by high-resolution X-ray diffraction (XRD, Smartlab RIGAKU) with a 2-bounce monochromator. We confirmed that all samples have a monodomain structure. The polarization direction of the BTO film was measured by piezoresponse force microscopy (PFM, MFP-3D Oxford instruments). The polarization direction in 5 and 15 nm BTO deposited on NSTO is headed to the substrate (down) (see Supplementary Figs. S1d, e online). 50 nm BTO deposited on SRO/LSAT has the opposite direction (up) owing to the difference of substrate. The characterization of thin films is described in Sections S1–S3 of the Supplementary Information.

### Measurement setup

We used the experimental equipment of AR-HAXPES at BL47XU in SPring-8^18^. Generally, the ionization cross-section decreases with increasing photon energy^37^. The required photon energy is estimated to be 8 keV when taking into account the escape depth of each atomic orbital. In the present study, a photon energy of 7.94 keV with a bandwidth of 38 meV was obtained using the Si (111) double monochromator and the Si (444) channel-cut monochromator. The X-ray beam was focused to 30 × 40 (samples without top electrode) or 1 × 5 μm^2^ (samples with Pt top electrode) regions on the sample surface. The AR-HAXPES apparatus installed in BL47XU has a wide-acceptance-angle objective lens ahead of the conventional HAXPES system with a hemispherical electron energy analyzer (R-4000-VG-Scienta Co.). The angle between the AR-HAXPES apparatus and photon propagation is fixed at 90° in all experiments. We show the optical configuration of our AR-HAXPES and discuss how to analyze AR-HAXPES data in Sections S4 and S5 of the Supplementary Information.

## Supplementary information

Supplementary information
