# Supplementary material for: Skewed electronic band structure induced by electric polarization in ferroelectric BaTiO3
Source: Sci Rep. 2020 Jul 1;10:10702. doi: 10.1038/s41598-020-67651-w (PMC7329818; doi:10.1038/s41598-020-67651-w)
Supplement: Supplementary file 1 — Supplementary information [file 41598_2020_67651_MOESM1_ESM.pdf]

Supplementary Information

Skewed electronic band structure induced by electric polarization in ferroelectric $BaTiO_3$

Norihiro Oshime[1*], Jun Kano[1,2,3†], Eiji Ikenaga[4], Shintaro Yasui[5], Yosuke Hamasaki[5], Sou Yasuhara[5], Satoshi Hinokuma[2,6], Naoshi Ikeda[1], Pierre-Eymeric Janolin[3], Jean-Michel Kiat[3], Mitsuru Itoh[5], Takayoshi Yokoya[7], Tatsuo Fujii[1], Akira Yasui[4], and Hitoshi Osawa[4]

[1]Graduate School of Natural Science and Technology, Okayama University, Okayama 700-8530, Japan. [2]Japan Science and Technology Agency, PRESTO, Kawaguchi, Saitama 332-0012, Japan. [3]Université Paris-Saclay, CentraleSupélec, CNRS, Laboratoire SPMS, 91190, Gif-sur-Yvette, France. [4]Japan Synchrotron Radiation Research Institute, JASRI, Sayo, Hyogo 679-5198, Japan. [5]Laboratory for Materials and Structures, Tokyo Institute of Technology, Yokohama 226-8503, Japan. [6]Innovative Oxidation Team, Interdisciplinary Research Center for Catalytic Chemistry, AIST, Tsukuba, Ibaraki 305-8565, Japan. [7]Research Institute for Interdisciplinary Science, Okayama University, Okayama 700-8530, Japan.

Correspondence to
*sc421235@s.okayama-u.ac.jp
†jun@psun.phys.okayama-u.ac.jp

1. X-ray diffraction patterns of thin films

Supplementary Figure S1a shows XRD θ-2θ patterns of BTO and ALO films with 5 nm thickness. Both films were epitaxially grown with a cube-on-cube relation between film and substrate; (001)BTO||(001)NSTO and (100)BTO||(100)NSTO, (001)ALO||(001)NSTO and (100)ALO||(100)NSTO, respectively. Rocking curves of both films measured at *002* BTO and *004* ALO are shown in Supplementary Fig. S1b and full-width at half-maximum (FWHM) values were 0.137° (blue dashed line) and 0.108° (black line). There were no secondary and no different orientation peaks in both films. The crystal mosaicities of both films were almost identical, as indicated by similar FWHM values.

2. Topographic and piezoresponse images

Supplementary Figure S1c shows the typical topographic image of BTO thin film measured together with PFM. BTO film has the very flat surface with roughness (Rms) of 0.2 nm. As shown in Supplementary Fig. S1d, positive 3 V writing on a 2 × 2 μm^2 area was

performed for poling treatment along substrate surface normal, then negative 3 V writing under 1×1 μm^2 at the center of a 2×2 μm^2 area was also performed. Additionally, the opposite bias for the same writing configuration was applied to the same BTO film, as shown in Supplementary Fig. S1e. Both the as-deposited area and the negative-bias writing area showed the same PFM phase contrast, although contrariwise, a positive bias writing area showed the opposite PFM phase contrast. No in-plane contribution of ferroelectricity was found by PFM measurement because this BTO film with the very thinner thickness was grown on NSTO substrate with the fully compressive strain from NSTO. The reason is that a lattice parameter of NSTO substrate is smaller than that of BTO. In the case of BTO, it is well known experimentally and theoretically that tetragonality is enhanced by compressive strain[1]. Therefore, BTO film with 5 nm thickness has only single *c*-domain and their polarization direction is headed to the substrate (down) (see Supplementary Figs. S1d, e). The thickness of 15 nm has the same direction. But 50 nm BTO deposited on SRO/LSAT has opposite direction (up) owing to the difference of substrate, which is related to the valance of band alignment between ferroelectric thin film and conductive substrate[2]. Note that, ALO thin film did not show any piezoresponse.

3. The possibility of polarization direction governed by the substrate termination

We consider the polarization direction into the ferroelectric thin film. Valence mismatch at the interface between ferroelectric thin film and conductive substrate gives good information. The valence mismatch so-called as polar discontinuity was firstly proposed for the interface of insulating materials such as $LaAlO_3/SrTiO_3$[3]. Since nominal ionic charges are relatively different, e.g. $(LaO)^+/(TiO_2)^0$ and $(AlO_2)^-/(SrO)^0$, characteristic electron transport exhibits at the interface. For heterointerfaces of ferroelectric-metallic substrate such $BiFeO_3$ (BFO)/SRO and BFO/$La_{0.7}Sr_{0.3}MnO_3$, the concept of polar discontinuity is also applicable for

the determination of polarization direction[4,5]. NSTO substrate with pretreatment of ultrapure water washing has a self-termination of TiO_2. Because hydroxylation reaction easily occurs on the surface of SrO resulting TiO_2 termination, similar to the case of wet etching in acid aqueous solution with pH 4.5[6]. SrO self-termination of the SRO buffer layer is formed owing to high volatile of RuO_2 layer during growth into the PLD chamber[7]. Supplementary Figure S2 shows the possible interface structure of our thin films, BTO/NSTO and BTO/SRO. Though BTO/NSTO interface has small valence mismatch due to the Nb doping, this value is almost negligible. So both thin films have no valence mismatch at the interface, indicating that a deterministic control over the polarization orientation using the concept of polar discontinuity is meaningless at least in BTO/NSTO and /SRO interfaces. As mentioned in Section 2, we experimentally confirmed the polarization direction using PFM. In this case, built-in field may determine the preferential direction of as grown sample[2].

4. Angle-resolved hard x-ray photoemission spectroscopy (AR-HAXPES)

The detailed experimental setup of angle-resolved hard x-ray photoemission spectroscopy (AR-HAXPES) at the BL47XU beamline in Spring-8 is described in ref. 8. The emission angle of photoelectrons depends on the escape depth as shown in Supplementary Fig. S3. The objective lens has a wide acceptance angle of 64°. Since angular resolution corresponds to depth resolution from the sample, photoemission detection by the objective lens produces a wide depth-dependent analysis with a resolution of 1.32^{o} even with a one-shot and fixed optical system. On the other hand, a conventional AR-HAXPES without a wide-angle objective lens is often required to mechanically adjust the optical angle between the incident beam and the sample, a feature causes difficulty in accurate angular-resolution and beam-positioning within the micrometer domain on samples. The energy resolution was estimated to be about 0.23 eV by Au Fermi-edge measurement.

We checked spectra with angle-integrated HAXPES in survey setting (TOA = 88.3^{o}) and then confirmed peak selection (Supplementary Fig. S4a), with the result that Ti-2$p_{3/2}$, O-1s, Ba-3$d_{5/2}$, and valence band in BTO and O-1s in ALO were selected. Then, we changed angular-resolved setting (TOA = 35°) and analyzed the observed atomic orbitals with the following process: (1) background was subtracted by Shirley method; (2) subtracted spectrum was fitted by Voigt function (see gray curves in the inset of Supplementary Fig. S3); (3) the binding energy of the atomic orbital was estimated to be the center position of FWHM. Finally, the depth-dependence of the energy shift in the atomic orbital was determined. Emission angles of 5° to 65° correspond to photoelectron emissions from the surface to a deeper region, respectively. As shown in spectra of ALO sample with 5 nm thickness (the inset of Supplementary Fig. S3), O-1s splits into two peaks: one in the vicinity of 532.5 eV and another in the vicinity of 530.6 eV. With increasing sample depth, the magnitude of the lower-energy peak increases gradually. So the higher- and the lower-energy peaks are assigned to ALO and NSTO, respectively. Indeed, the signal detection at different emission angle is a good idea for distinguishing surface, bulk and interface binding energies, but we have to accurately consider a depth sensitivity. In the spectrum of ALO at emission angle = 35° corresponding to the bulk state, the contribution derived from substrate already appears. We calculated inelastic mean free path (IMFP) of the electrons at 7.94 keV photon energy as in Supplementary Fig. S4b. TPP-2M formula proposed in ref. 9 was applied in the present study. Theoretically calculated IMFPs for BTO are around 9.5 nm (Supplementary Fig. S4b), hence the effective probing depth d_p is some 19 nm. This d_p is empirically estimated in AR-HAXPES at the BL47XU beamline. Since signal intensity is attenuated by exp(-d_p/λ) where λ is the IMFP, signal at $d_p \sim 3\lambda$ has less than 5% intensity. Therefore, the signal at low emission angle mainly comes from the surface region, but the signal at high emission angle integrates over the whole film in the case of BTO with 5 nm thickness as illustrated in Supplementary Fig. S4c. When the electric polarization points into the substrate

(down), it is theoretically expected that the binding energy increases from surface to interface region[10]. If the actual energy shift shows small, measured binding energy does not show any shift owing to the relative weighting of deeper regions at high emission angle. Our experimental results clearly show the binding energy shift (see Fig. 1a, b in the manuscript). Actual skewed band structure of BTO with 5 nm thickness has steep shifts of the binding energy compared with the experimental values. The depth sensitivity mentioned above will affect at 15 nm thickness BTO. The effect is negligible in the BTO with 50 nm thickness. We concluded that electronic states of each atomic configuration in heterostructure can be thus observed with depth-resolved photoemission signal.

No extrinsic charging effect was observed in our experimental data. Such a charge is often seen in photoemission experiments, where it degrades the accuracy of the data. In an extrinsic charge situation, the energy level of photoelectrons at the surface is often altered by the electric field of the surface space charge, expanding the distribution of kinetic energy and resulting in a spectral profile that increases in width as the depth decreases towards the surface. However, we confirmed that the Gaussian width of the Voigt function was almost constant with respect to the emission angle, i.e. the distribution of kinetic energy was not expanded. Thus we conclude that there was no surface charge on our samples in the present study. Electron beam irradiation by the flood gun was not used, to avoid spectral distortion. Also, the effect of surface photovoltage can be escaped because the photon flux of 2.3×10^{16} photons/(cm^2 s)[8] is enough low.

5. AR-HAXPES with polarization switching

Polarization switching of 50 nm sample has been done using a function generator (WF1947, NF corp.) with the current-voltage (*I*-*V*) curves monitoring[11]. The function generator contacted to the bottom electrode (SRO) by silver wire with a 20 μm diameter. A frequency

range for an ac voltage was 500 kHz with an amplitude of 6 V_{pp} (peak-to-peak voltage). When we carried out the AR-HAXPES measurement for polarization pointing up (an inherent direction of the polarization of 50 nm thickness sample heads to the surface), a top electrode (Pt) was connected to the bottom electrode (SRO) forming a short-circuited. For polarization pointing down, 3 cycles ac voltage was applied then a dc voltage of -2 V was applied to the sample. -2 V nearly corresponds to a coercive field of 50 nm sample. Since the polarization backswitching is difficult to keep long retention caused by the imprint causing the retention loss[12,13], applied dc voltage was kept during AR-HAXPES measurement. Note that applied dc voltage during the AR-HAXPES measurement does not affect the data collection. Because we confirmed no-spectral distortion due to the charging effect in every measurements (see the last paragraph of Sec. 4 of Supplemental Information).

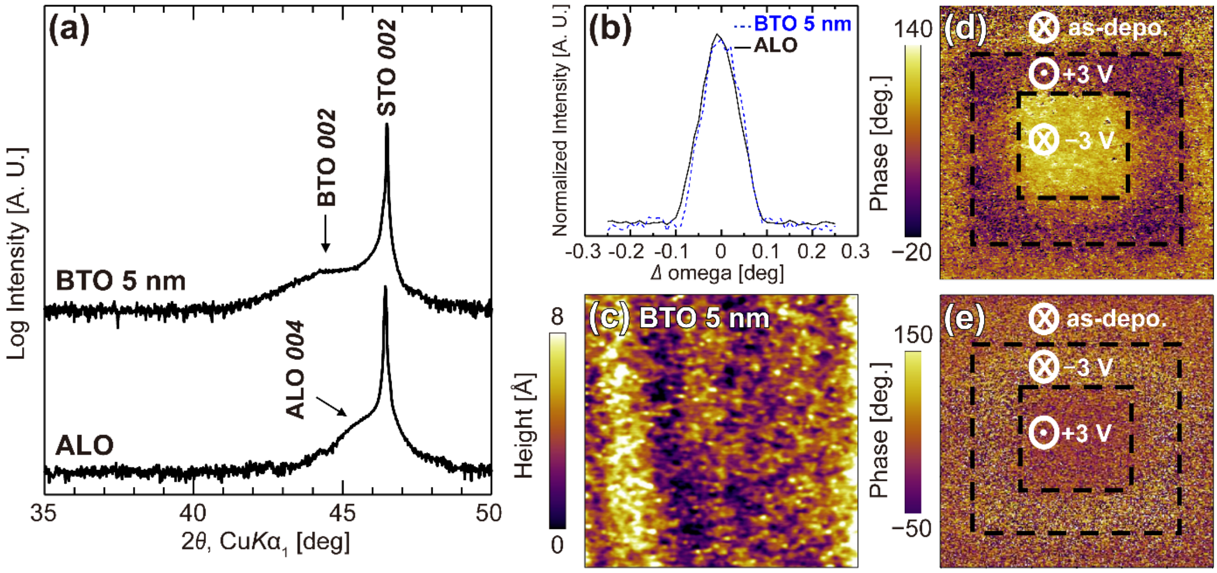

Supplementary Figure S1. (a) XRD patterns of BTO and ALO films with 5 nm thickness on NSTO substrates. (b) Rocking curves measured at BTO *002* (blue dashed line) and ALO *004* (black line) diffractions. (c) Topographic image of the BTO film. Piezoresponse phase images of BTO film: (d) +3 V (2 × 2 μm^2 area, outside) and -3 V (1 × 1 μm^2 area, inside) and (e) -3 V (2 × 2 μm^2 area, outside) and +3 V (1 × 1 μm^2 area, inside) writing treatments, with a measured area of 3 × 3 μm^2. Light and dark regions correspond to negative and positive polarization directions, respectively.

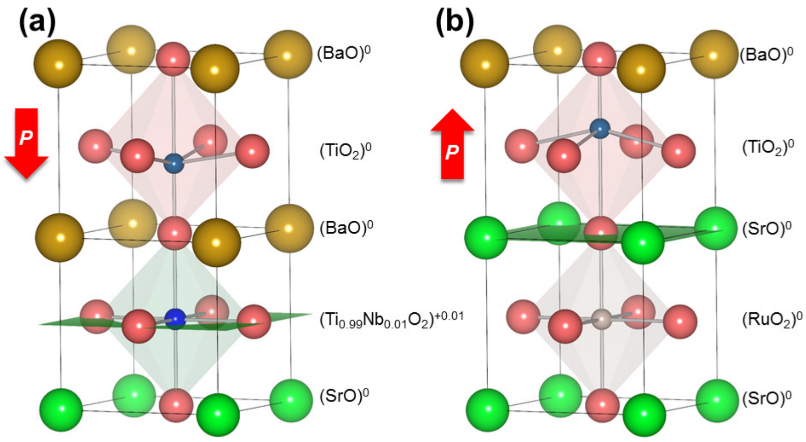

Supplementary Figure S2. Schematic picture of possible interface structure of our thin films. (a) BTO/NSTO, (b) BTO/SRO. Each stacking layer in BTO/NSTO describes BaO-TiO_2-BaO-$Ti_{0.99}Nb_{0.01}O_2$-SrO. BaO-TiO_2-SrO-RuO_2-SrO is for BTO/SRO deposited on LSAT substrate. Green (100) lattice plane indicates the termination of substrate. Superscript number is a nominal ionic charge. These schematics were drawn by VESTA[14].

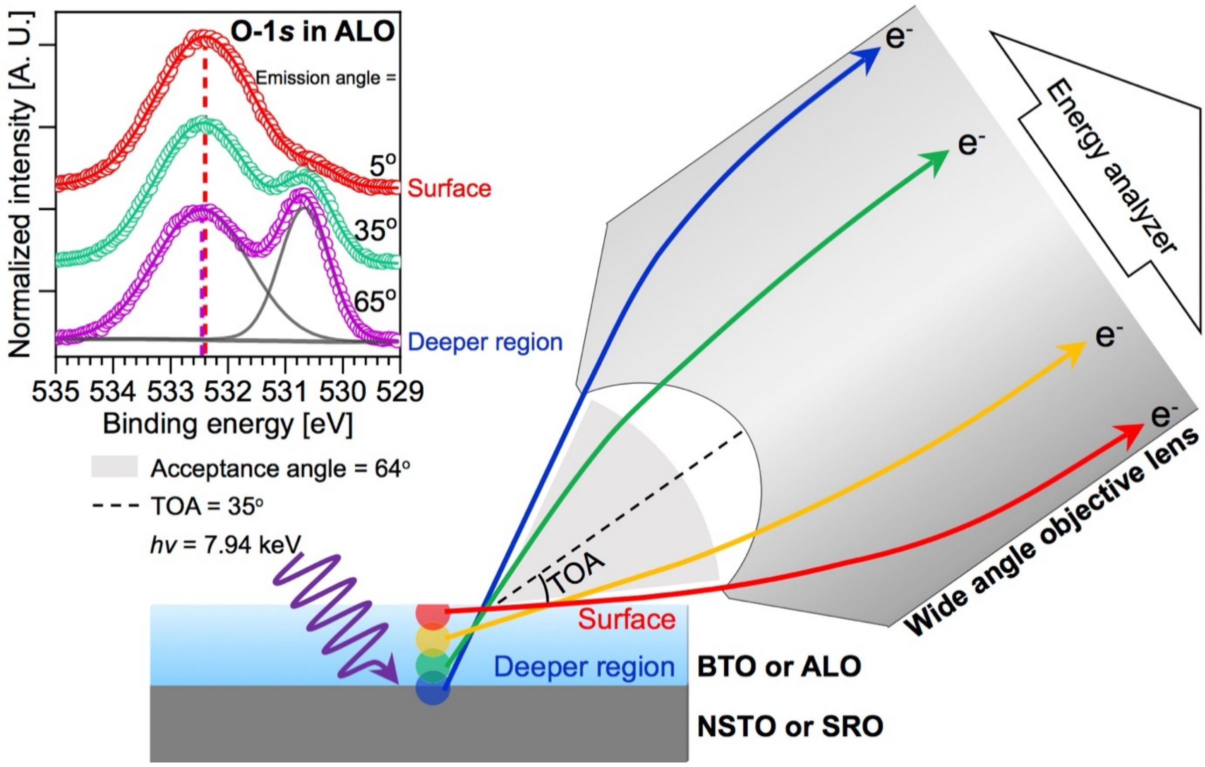

Supplementary Figure S3. Schematic picture of AR-HAXPES with wide-angle objective lens for BTO (or ALO) thin film. The angle between the AR-HAXPES apparatus and incident beam is fixed at 90° in all experiments. The lens has a 64° acceptance angle. Take-off angle (TOA) is defined as the angle between the sample surface and the lens. The emission angle of photoelectrons increases with increasing escape depth, their TOA was determined to be 35°. The inset shows AR-HAXPES spectra of O-1*s* in ALO. The probing depth in photoemission increases as the emission angle increases. Red and purple circles are surface and deeper regions, respectively. In the spectrum at emission angle = 65°, curves of background and Voigt function are drawn as gray lines.

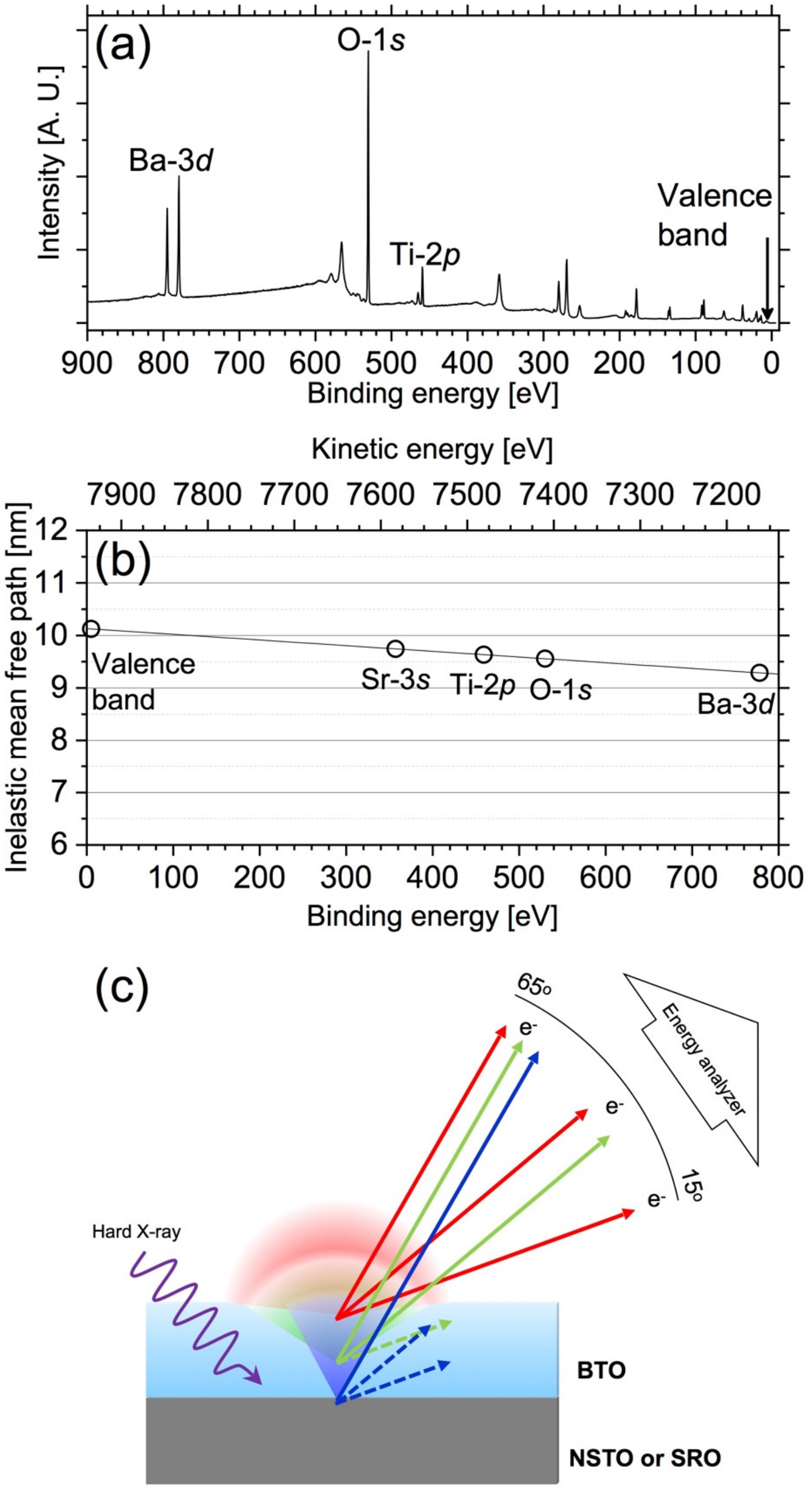

Supplementary Figure S4. (a) Survey HAXPES spectrum of BTO with 5nm thickness. TOA is fixed at 88.3°. (b) Calculated IMFP of atomic orbitals and valence band of BTO using TPP-2M formula. (c) Schematic picture of depth sensitivity problem in AR-HAXPES.

References

[1] Choi, K. J. *et al.* Enhancement of ferroelectricity in strained $BaTiO_3$ thin films. *Science* **306,** 1005-1009 (2004).

[2] Lichtensteiger, C., Fernandez-Pena, S., Weymann, C., Zubko, P., & Triscone, J. -M. Tuning of the depolarization field and nanodomain structure in ferroelectric thin films. *Nano Lett.* **14,** 4205-4211 (2014).

[3] Ohtomo, A. & Hwang, H. Y. A high-mobility electron gas at the $LaAlO_3/SrTiO_3$ heterointerface. *Nature* **427,** 423-426 (2004).

[4] Yu, P. *et al.* Interface control of bulk ferroelectric polarization. *PNAS* **109,** 9710-9715 (2012).

[5] De Luca, G. *et al.* Nanoscale design of polarization in ultrathin ferroelectric heterostructures. *Nat. Commun.* **8,** 1419; 10.1038/s41467-017-01620-2 (2017).

[6] Kawasaki, M. *et al.* Atomic control of the $SrTiO_3$ crystal surface. *Science* **266,** 1540-1542 (1994).

[7] Rijnders, G., Blank, D. H. A., Choi, J., & Eom, C.-B. Enhanced surface diffusion through termination conversion during epitaxial $SrRuO_3$ growth. *Appl. Phys. Lett.* **84,** 505-507 (2004).

[8] Ikenaga, E. *et al.* Development of high lateral and wide angle resolved hard x-ray photoemission spectroscopy at BL47XU in SPring-8. *J. Electron Spectrosc. Relat. Phenom.* **190,** 180-187 (2013).

[9] Tanuma, S., Powell, C. J., & Penn, D. R. Calculations of electron inelastic mean free paths. V. Data for 14 organic compounds over the 50–2000 eV range. *Sur. Interface Anal.* **21,** 165-176 (1994).

[10] Liu, X., Burton, J. D., & Tsymbal, E. Y. Enhanced tunneling electroresistance in ferroelectric tunnel junctions due to the reversible metallization of the barrier. *Phys. Rev. Lett.* **116,** 197602 (2016).

[11] Federicci, R. *et al.* $Rb_2Ti_2O_5$: Superionic conductor with colossal dielectric constant. *Phys.*

Rev. Mater. **1,** 032001(R) (2017).

[12] Scott, J. F. *Ferroelectric Memories*. (Springer-Verlag, Berlin, Heidelberg, 2000).

[13] Kang, B. S. *et al.* Mechanisms for retention loss in ferroelectric Pt/Pb($Zr_{0.4}Ti_{0.6}$)O_3/Pt capacitors. *Appl. Phys. Lett.* **82,** 2124-2126 (2003).

[14] Momma, K. & Izumi, F. *VESTA 3* for three-dimensional visualization of crystal, volumetric and morphology data. *J. Appl. Crystallogr.* **44,** 1272-1276 (2011).
